# A descriptive assessment of a broad range of food-related parenting practices in a diverse cohort of parents of preschoolers using the novel Real-Time Parent Feeding Practices Survey

**DOI:** 10.1186/s12966-022-01250-y

**Published:** 2022-03-02

**Authors:** K. A. Loth, Z. Ji, J. Wolfson, D. Neumark-Sztainer, J. M. Berge, J. O. Fisher

**Affiliations:** 1 Department of Family Medicine and Community Health, University of Minnesota Medical School, Minneapolis, MN USA; 2 Division of Biostatistics, School of Public Health, University of Minnesota, Minneapolis, MN USA; 3 Division of Epidemiology and Community Health, School of Public Health, University of Minnesota, Minneapolis, MN USA; 4 College of Public Health, Temple University, Philadelphia, PA USA

**Keywords:** Food parenting practices, Eating patterns, Dietary intake, Ecological momentary assessment, Real-time assessment

## Abstract

**Background:**

Much of the research to-date on food parenting has evaluated typical use of various parent feeding practices via questionnaire. *The Real-Time Parent Feeding Practices* Measurement survey was developed for use within an Ecological Momentary Assessment (EMA) protocol to capture momentary use of parent feeding practices in real-time.

**Methods:**

This manuscript describes the development of the EMA-based *Real-Time Parent Feeding Practices *survey and highlights initial descriptive data on the real-time use of 22 individual parent feeding practices (e.g., pressure-to-eat, guided choices, etc.) as reported via EMA by parents of preschool-aged children (*n* = 116) over a 10-day data collection time period. A total of 3382 eating occasions were reported, with an average of 29.2 reported eating occasions per participant.

**Results:**

Results revealed that most participants used a variety of food-related parenting practices day-to-day that span four higher-order domains: structure, autonomy support, coercive control and indulgence. Supportive feeding practices, defined as those from the structure and autonomy support domains, were reported most frequently, with one or more structure behavior (e.g., specific mealtime rules/routines) was used at 88.9% of reported eating occasions and one or more autonomy support behavior (e.g., involvement of the child in meal preparation) was used at 87.3% of eating occasions. While unsupportive feeding practices, defined as practices from within the coercive control (e.g., pressure-to-eat) and indulgent (e.g., anticipatory catering) feeding domains, were reported less frequently, one or more behaviors from each of these domains were still reported at over 25% of all eating occasions.

**Conclusions:**

Results of the current study take a next step towards deepening our understanding of the use of a broad range of food-related parenting practices in real-time. Findings revealed that the vast majority of practices used by parents fall within the structure and autonomy support domains. However, most parents did not exclusively use supportive or unsupportive practices, rather they used a combination of food-related parenting practices across all domains. Future research should continue to explore a broad range of food-related parenting practices and seek to understand how parent approaches to feeding are associated with long-term child outcomes, including dietary intake, food preferences, and eating patterns.

## Introduction

Children’s eating behaviors, dietary intake and weight status are shaped significantly by the socialization that occurs in the family and home food environment [1–3]. Specifically, parents influence their children’s eating their use of food-related parenting practices [4, 5], or goal-oriented actions and behaviors that parents use to shape their children’s eating behaviors or dietary intake. Current theoretical frameworks of food-related parenting practices describe three overarching domains of feeding practices: structure, such as food availability and limit setting; autonomy support, such as praise and reasoning; and coercive control, such as pressure-to-eat and overt food restriction [1, 6]. Indulgence has been discussed as a both a sub-domain of structure [1], as well as proposed to be a fourth unique high-level domain of potential importance [4]; indulgent behaviors allow the child complete freedom over what, when, and/or how much to eat as well as involve catering to the child’s preferences to avoid power struggles over food. Studies to date have generally found that practices that provide structure (e.g., the availability of healthful foods in the home, limit setting) and autonomy support (e.g., reasoning, encouragement) for children’s eating have positive associations with healthful dietary intake (e.g., fruit and vegetables) and eating behaviors. Alternatively, coercive (e.g., pressure to eat) and indulgent practices (e.g., feeding different foods at meals than the rest of the family) have been associated with less healthful dietary intake (e.g., sugar sweetened beverages), a higher body mass index, and the development of maladaptive eating behaviors over time [2, 3, 7–9]. While research to date generally points to a supportive role of structure and autonomy supportive practices for the development of healthy eating behaviors and weight among children, evidence of benefits for specific practices remains limited [1–3, 7].

While feeding interactions are thought be dynamic and sensitive to context, the vast majority of research has evaluated parents’ “usual” use of specific food-related parenting practices via questionnaire or practices at a specific point in time using meal observations. Both approaches fail to account for potentially important within- or between-day variation across time and contexts. Indeed, our recent qualitative research revealed that parents use of specific feeding approaches with their preschool aged children varied both within meals and between meals in response to a range of situational or “momentary” influences including parent and child mood, external time pressures (e.g., work schedule changes, outside activities), and child behavior [4]. In particular, parents described shifts away from structure and autonomy supportive practices to more indulgent and controlling practices in response to situational influences. For instance, Berge and colleagues captured quantitative evidence of these momentary shifts in food parenting practices using Ecological Momentary Assessment (EMA) within a racially/ethnically and socioeconomically diverse sample of school-aged children and their parents; they found that higher levels of parental stress or depressive symptoms experienced earlier in the day were associated with the use of more controlling food-related parenting practices later in the day [10, 11]. Taken together, these studies highlight that traditional approaches to studying food parenting may not capture important variation across time and context in the way parents approach feeding their children. For example, a parent might have the overarching goal of promoting healthful dietary intake for their child which leads them to rely on structure and autonomy support food-related parenting practices at the majority of meals; however, when faced with their child’s refusal to eat vegetables at a particular meal, they shift away from their usual feeding approach to use of coercive/controlling practices (e.g., pressure-to-eat) at that particular meal, while keeping the same overarching goal of healthful dietary intake. EMA can be used to characterize the frequency, variety and patterning of food-related parenting practices, providing insights into real-time use of feeding practices and their relationship with dynamic momentary influences, such as mood, stress and context.

This research was undertaken to capture momentary use of a broad range of food-related parenting practices in real-time via the EMA-based *Real-Time Parent Feeding Practices* survey, developed for this study. The current study presents the development of and initial descriptive data from a 10-day EMA protocol assessing parents’ use of 22 individual food-related parenting practices (e.g., pressure-to-eat, guided choices, etc.) along higher-order dimensions of structure, autonomy support, coercive control, and indulgence in an ethnically and socio-economically diverse sample of parents (*n* = 116) of preschool-aged children. Results from the current study represent a crucial next step in deepening our understanding of the momentary or contextual influences on the goal-directed practices used by parents to feed their children and highlight the methodological utility of assessing practices at the meal-level, rather than based on parent-report of “usual” practices.

## Methods

Data for the present study are from Kids EAT!, mixed-methods observational study designed to deepen our understanding of parents’ experiences feeding their preschool-aged child and the factors that influence their choices about feeding [12]. Kids EAT! study participants (*n* = 116) completed traditional questionnaires about demographics, family routines and functioning, and child feeding and eating behaviors via online surveys, followed by 10 days of ecological momentary assessment (EMA) completed via cell phone during the fall of 2019. The current study only uses data from the EMA data collection protocol.

### Study population, recruitment and participant demographics

**Fig. 1:**
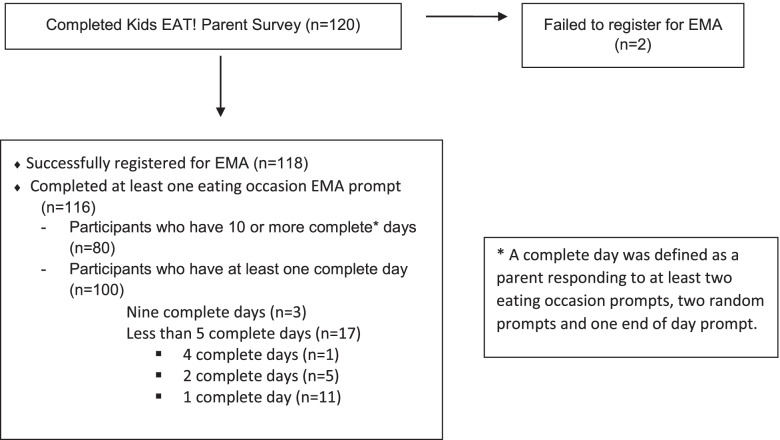
Kids EAT! Research Study Participant Engagement Details

Kids EAT! is an ancillary study to EAT 2010–2018 (Eating and Activity among Teens) a large, population-based cohort study on eating and weight-related health [13]. Survey data collected from 1491 young adults (Mean age 22.2) as a part of EAT 2018 were utilized to identify potential Kids EAT! participants that met the inclusion criteria; young adults who indicated on the EAT 2018 survey that they had at least one child aged 2–5 years who lived with them at least 50% of the time were invited by email to participate in the Kids EAT! study. Participants in the original EAT 2010–2018 cohort lived in the Minneapolis – Saint Paul metropolitan area during their initial participation in 2010; eligible participants were invited to participate in Kids EAT! regardless of their current geographic location at the time of data collection for this study. Kids EAT! recruitment e-mails indicated that the study goal was to learn more about parents’ experiences feeding their pre-school aged child and provided some information about study data collection procedures. Figure 1 describes the degree of engagement of participants from completion of the Kids EAT! baseline study survey through participation in the EMA protocol, including highlighting participants who dropped out of the study. The University of Minnesota’s Institutional Review Board Human Subjects Committee approved all protocols used for the Kids EAT! study.

Table 1 provides demographic information on the sample. The participating parents (*n* = 116) had a mean age of 26.3 at the time of survey completion. The majority (81.9%) of parent participants were female. Just over half of participants (56%) reported education beyond high school. Approximately 24% of the sample reported household incomes below the 2020 Federal Poverty line for household sizes of 2 or more individuals ($17,240). Child gender was roughly evenly split between male and female children.

**Table 1:** Study demographic characteristics (*n* = 116)

	n (%)
Parent Gender	
Female	95 (81.9)
Male	21 (18.1)
Parent Race/Ethnicity (%)	
Black	39 (33.6)
Hispanic	26 (22.4)
Asian	20 (17.2)
White	17 (14.7)
More than One Race/Other	7 (7.8)
Native American	5 (4.3)
Parent Education (%)	
Partial high school or less	11 (9.5)
High school graduate or GED	40 (34.5)
Partial college or specialized training	42 (36.2)
College graduate	20 (17.2)
Graduate degree	3 (2.6)
Spouse Education (%)	
Partial high school or less	11 (9.5)
High school graduate or GED	36 (31.0)
Partial college or specialized training	22 (19.0)
College graduate	10 (8.6)
Graduate degree	5 (4.3)
No spouse/not applicable	32 (27.6)
Household Income (%)	
$0–$9999	19 (16.4)
$10,000–$14,999	7 (6.0)
$15,000–$24,999	21 (18.1)
$25,000–$34,999	22 (19.0)
$35,000–$49,999	18 (15.5)
$50,000–$74,999	20 (17.2)
$75,000-and above	9 (7.8)

### Procedures and data collection

First, using an individualized link included in the study recruitment e-mail, participants completed the Kids EAT! baseline surveys online (not included in this report) on a wide range of topics including demographics, family routines and functioning, and child feeding and eating behaviors. Next, parents were given detailed instructions for how to complete the EMA protocol. The 10-day EMA data collection period began the day following survey completion. Standardized EMA data collection protocols from prior studies [13] were used to guide the development of EMA-based *Real-Time Feeding Practices* survey and sampling methods.

During the EMA data collection period, parents were asked to complete surveys in response to three types of EMA sampling methods: (1) signal (researcher initiated surveys at random times of day), 2) event contingent EMA recordings (participant initiated surveys following an eating occasion), (2) end-of-day surveys. The current study only uses data from event contingent recordings; data from all participants who recorded at least one eating occasion during the data collection period was included. Event contingent recordings were self-initiated by parents whenever the child ate in the presence of the parent (i.e., both meals and snacks); importantly, parents did not need to be sitting and eating with the child to complete a recording, they were only required to be present to the degree that they felt they could respond to the questions specific to the eating occasion. Event-contingent recordings asked parents to report on details of the eating occasion that prompted the recording, including their use of specific food-related parenting practices. Specific measures used in analysis for the current study are described in detail below. Parents completed these EMA recordings using their own electronic device (i.e., cell phone, tablet). On average, each EMA recoding took participants 2–3 min to complete.

Surveys were completed in English; participants’ English language fluency was determined during their initial enrollment in the EAT 2010–2018 study. Families were offered an incentive of a $150 gift card for participation in the Kids EAT! Study. Data collection was completed on all participants between October 2020 and February 2021.

### Measures

#### Real-time parent feeding practices survey

The EMA-based survey was developed for the current study to measure a broad range of food-related parenting practices within an EMA protocol. The survey includes 22 questions on food-related parenting practices situated within four higher level theoretical domains, including Coercive Control (5 items), Indulgent (3 items), Structure (5 items), Autonomy support (9 items); the language for each individual measure is included in Table 2. Individual questions were designed to measure specific sub-constructs as outlined in Vaughn’s content map of fundamental constructs in food parenting practices [1], drawing from existing questionnaires where possible, such as the Child Feeding Questionnaire [14] and the Food Parenting Inventory [15], and adapted for use within an EMA protocol. For example, an item on the Child Feeding Questionnaire designed to measure parental pressure to eat reads, “I have to be especially careful to make sure my child eats enough”. This question was adapted for the current study to focus on a parent’s specific behavior at the most recent meal or snack consumed by their child. The adapted question read, “Thinking of this meal or snack, did you have to encourage your child to eat more food than they wanted to?”. Parents responded yes/no for each item.

**Table 2:** Use of individual food-related parenting practices and overarching feeding domains across all reported meals and snacks within a sample of racially/ethnically and socioeconomically diverse 2–5 year old children

High Level Feeding Domain	Individual items from the *Real-Time Parent Feeding Practices* Survey ***Thinking about this meal or snack, did you … .***	Meals/snacks where behavior was endorsed % of total meals (N meals)
Use of any food parenting practice from the structure domain		88.85% (3005)
	sit and eat with your child	76.29% (2580)
	choose where your child ate the meal or snack	61.89% (2093)
	choose what foods your child got to eat	44.47% (1504)
	closely monitor the type and amount of food eaten by your child	41.13% (1391)
	allow your child to choose what to eat, from several options you had already picked out	27.38% (926)
Use of any food parenting practice from the autonomy support domain		87.29% (2952)
	involve your child in deciding what foods they would eat	65.70% (2222)
	allow your child to take seconds if they asked for them	65.23% (2206)
	teach your child about why you wanted them to eat more of certain foods	23.86% (807)
	teach your child about why you wanted them to eat less of certain foods	19.69% (666)
	tell your child you wanted them to eat more of certain foods	19.66% (665)
	encourage your child to try at least a small amount of all foods offered	19.31% (653)
	negotiate with your child about how much food they needed to eat	14.84% (502)
	negotiate with your child about what foods they needed to eat	12.60% (426)
	tell your child you wanted them to eat less of certain foods	11.47% (388)
Use of any food parenting practice from the coercive control domain		28.86% (976)
	have to encourage your child to eat more food than they wanted to	17.27% (584)
	offer your child a treat or reward for eating more	10.35% (350)
	have to make sure your child did not eat too much food	10.17% (344)
	offer your child a treat or reward for trying a new food	9.91% (335)
	trick or bribe your child into eating more than they wanted to	8.22% (278)
Use of any food parenting practice from the indulgent domain		27.56% (932)
	choose to prepare separate food that knew your child would enjoy eating	16.20% (548)
	allow your child to choose a separate meal or different food because they did not want to eat what was offered	14.99% (507)
	give your child food in order to calm them down or help manage their behavior	7.57% (256)

Note: There are 3382 total meals reported by the 116 participants, 29.16 total meals reported per participant on average, and 2.67 meals reported per participant per day on average. (Note that the data in this table are general statistics: number of meals where behavior was endorsed/total number of meals, not on participant-level or daily-level)

### Data analysis

**Fig. 2:**
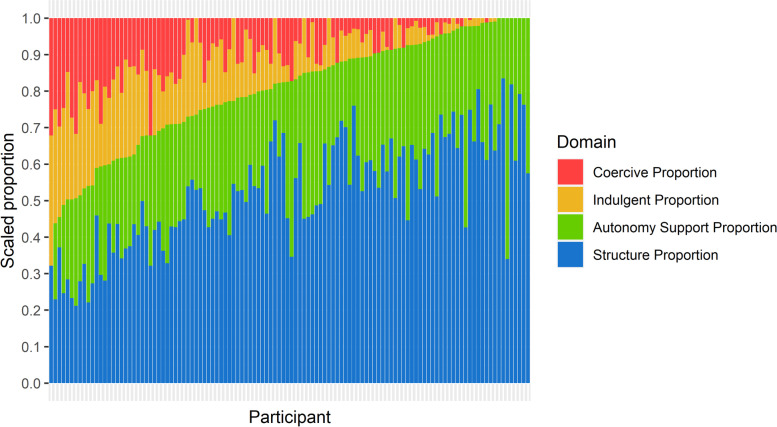
Scaled proportions of food-related parenting practices by domain and participant. Note: Each narrow column depicted in this figure helps to illustrate the proportion of food parenting practices across the four domains (each column totals to 100% of practices used), scaled by number of items in each domain, for one parent-child dyad in the current sample. For example, for the participant depicted in the column on the very far left side of the figure, food-related parenting practices from the structure domain represents 32.1% (0.32) of all food-related parenting practices used, scaled by the number of items included in structure domain; food-related parenting practices from the indulgent domain represents 35.7% (0.36) of all practices used scaled by the number of items included in indulgent domain; and food-related parenting practices from the indulgent domain represent 32.1% (0.32) of all practices used, scaled by the number of items included in coercive domain. Participants are presented in descending order of the sum of scaled coercive and indulgent proportion

The proportion of eating occasions where each individual food-related parenting practice was endorsed was calculated using the count of the total number of eating occasions in which the behavior was reported (answering ‘yes’ in the corresponding question), divided by the total number of eating occasions. The proportion of eating occasions where a certain feeding domain (e.g., structure, coercive control) was endorsed was calculated using the count of the total number of eating occasions which have at least one behavior from the domain reported (answering ‘yes’ in any of the corresponding questions of the domain) divided by the total number of eating occasions. Finally, the scaled proportion shown in Fig. 2 demonstrated the proportions of the food parenting practices that belong to each domain for every individual. The proportions were then scaled by the corresponding number of items in the domain. A sensitively analysis was conducted to examine for potential differences in use of specific food parenting practices (both individual and within domain) by number of data collection days completed (10 or more days vs less than 10 days). This sensitivity analysis revealed some heterogeneity in reported use of specific food parenting practices within the study population related to number of days participants engaged in data collection; however, there was overall homogeneity between the two groups at the domain level. Because the number of participants reporting less than 10 days was so small (representing less than 6.8% of all reported eating occasions) the eating occasions reported by this group were found to have a relatively small impact on overall results. Thus, in an effort to minimize selection bias in our sample, data from all participants were included in the final analytic sample. All data processing and analysis procedures were completed using R Version 4.0.2.

## Results

**Fig. 3:**
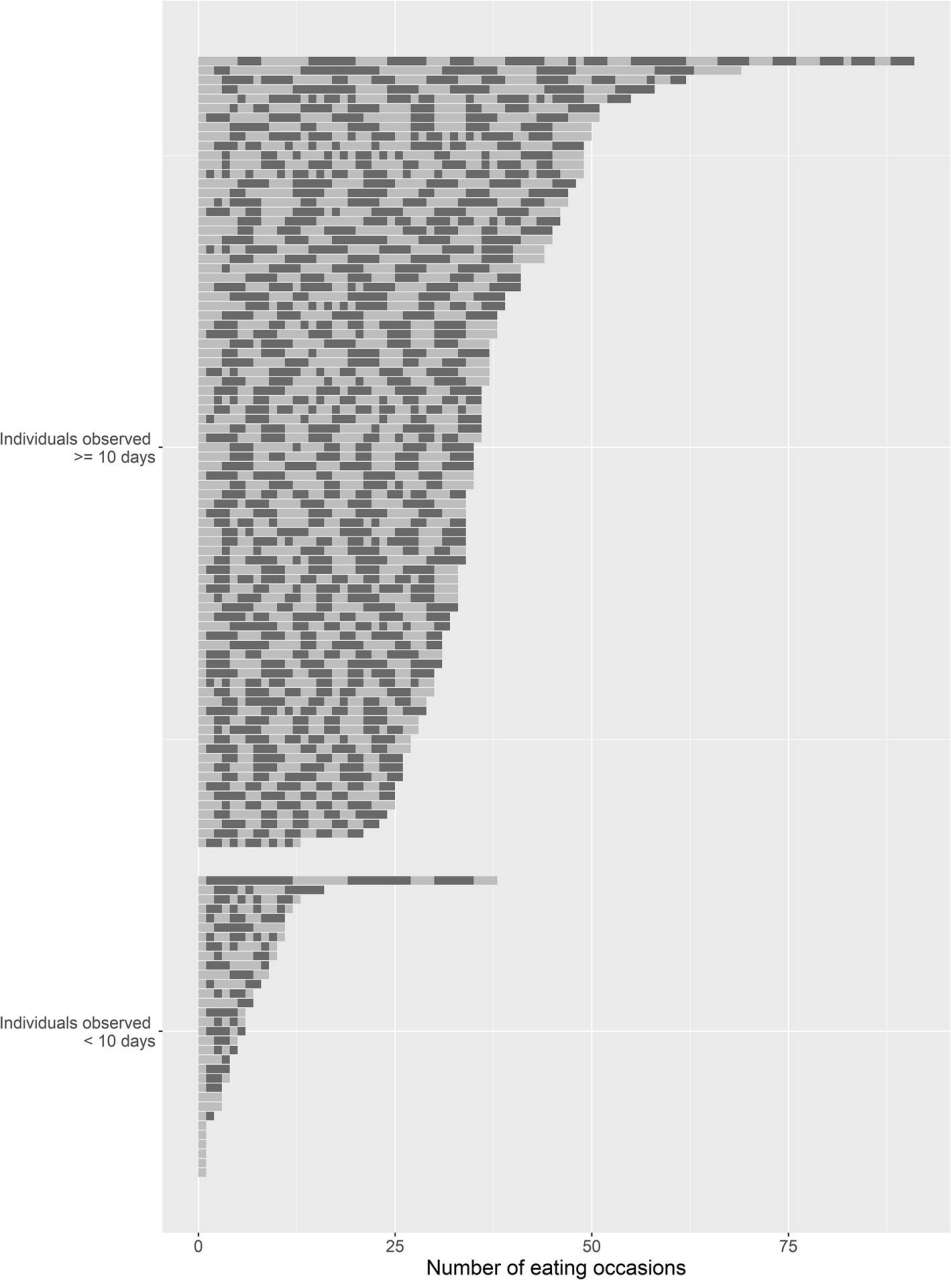
Total number of responses to eating occasion EMA prompts for each participant in the current study. Note: Number of event-contingent EMA-based Real-Time Parenting Practices surveys completed by participant and by number of EMA protocol days completed. The lighter and darker colors indicate different days throughout the study period. A total of 84 out of 116 parents provided 10 or more days of EMA data using the Real-Time Parenting Practices survey. On average, 29.2 eating occasions were reported per participant over the data collection period with a mean of 2.7 eating occasions reported per participant per day

A total of 3382 eating occasions were reported by participants over the 10-day EMA data collection period. On average, 29.2 eating occasions were reported per participant with a mean of 2.7 eating occasions reported per participant per day. Fig. 3 provides detailed information on number of responses to eating occasion EMA prompts at the participant level.

### Structure

Structure feeding practices were reported at the vast majority (88.9%) of all recorded eating occasions. The structure feeding practice reported at the highest percent of eating occasions was parents sitting and eating with their child; this practice was reported by parents at 76.3% of eating occasions. Parents reported choosing where their child ate meals and snacks at 61.9% of eating occasions; choosing what foods their child got to eat at 44.5% of eating occasions; closely monitoring the type and amount of food eaten by their child at 41.1% of eating occasions; and allowing their child to choose what they wanted to eat, from several options you had already picked out (e.g., guided choices) at 27.4% of eating occasions.

### Autonomy support

Autonomy support feeding practices were reported at 87.3% of all recorded eating occasions. The autonomy support practice reported at the highest percent of eating occasions was parents indicating that they involved their child in deciding what foods they would eat; this practice was reported at 65.7% of eating occasions. Similarly prevalent was parents allowing their child to take “seconds” at a meal if they wanted them; this behavior was reported by parents at 65.2% of eating occasions. Parents reported teaching their child about why they wanted them to eat more of certain foods at 23.9% of eating occasions; teaching their child about why they wanted them to eat less of certain foods at 19.7% of eating occasions; telling their child they wanted them to eat more of certain foods at 19.7% of eating occasions; encouraging them to try at least a small amount of all foods offered at 19.3% of eating occasions; negotiating with their child about how much food they needed to eat at 14.8% of eating occasions; negotiating with their child about what foods they need to eat at 12.6% of eating occasions, and telling their child they wanted them to eat less of certain foods at 11.5% of eating occasions.

### Coercive control

Coercive control feeding practices were reported at around one quarter (28.9%) of all recorded eating occasions. The coercive feeding practice reported at the highest percent of eating occasions was parents encouraging their child to eat more than they wanted to eat; this behavior was reported at 17.3% of all recorded eating occasions. Parents reported offering their child a treat or reward for eating more food at 10.4% of eating occasions and offering a treat or reward for trying a new food at 9.9% of eating occasions; and having to make sure their child did not eat too much food at 10.2% of eating occasions. Finally, parents reported tricking or bribing their child into eating more food than they wanted to at 8.2% of all recorded eating occasions.

### Indulgent

Indulgent feeding practices were reported at around one quarter (27.6%) of all recorded eating occasions. The indulgent feeding practice reported at the highest percent of meals was parents choosing to prepare separate foods that they knew their child would enjoy eating; this behavior was reported at 16.2% of all recorded eating occasions. Parents reported allowing their child to choose a separate meal or different food because the child did not want to eat what was offered at 15.0% of recorded eating occasions; and giving their child food in order to calm them down or help to manage their behavior at 7.6% of eating occasions.

### Proportion of behaviors used by domain

As illustrated in Fig. 2, the scaled proportion of nearly all families’ use of structure (e.g., mealtime rules and routines) and autonomy support (e.g., guided choices, nutrition education) feeding practices represented the greatest proportion of all practices used. Since separate proportions were calculated for each family, these proportion percentages are described in ranges. On average, for the participating families, structured practices represented 52.6% (Range: 22.1 to 83.5%) of all practices used, scaled by the number of items categorized as structured practices. Autonomy supportive practices represented 27.6% of all practices used (Range: 12.9 to 55.0%) of all practices used, scaled by the number of items categorized as autonomy supportive practices. Similarly, coercive practices represented 9.1% (Range: 0.0 to 29.0%) of all practices used after scaling, and indulgent practices represented 10.7% (Range: 0.0 to 26.8%) of all practices used after scaling. Importantly, nearly all families included in the sample used a combination of feeding practices across all four domains.

## Discussion

The current study research describes the development of and initial descriptive data from the newly developed EMA-based *Real-Time Parent Feeding Practices* survey; this tool allows for assessment of the real-time use of 22 individual food-related parenting practices (e.g., pressure-to-eat, guided choices, etc.) within an EMA protocol. Findings revealed that most parents of preschoolers use a variety of food-related parenting practices day-to-day that span all four higher-order domains of coercive control (e.g., restriction, pressure-to-eat), indulgence (e.g., anticipatory catering, child choosing meals), structure (e.g., mealtime rules and routines), and autonomy support (e.g., guided choices, nutrition education). Structure and autonomy support behaviors were reported most frequently; one or more structure behavior was used at the majority of reported meals and one or more autonomy support behavior was used at the majority of meals. While coercive control and indulgent behaviors were reported less frequently, one or more behaviors from each of these domains were still reported at more than one quarter of all eating occasions. Collectively, these results highlight the utility of capturing data on food-related parenting practices in real-time and suggest that, 1) use of supportive (i.e. structure and autonomy support) and unsupportive (i.e., coercive control and indulgent) practices are not mutually exclusive – most parents use a wide range of practices, and 2) the majority of food-related parenting practices used by most parents are supportive practices, falling within the structure and autonomy support domains. Importantly, parents of preschoolers in our sample were able and willing to engage in a 10-day EMA study protocol that required them to report on their use of food-related parenting practices at multiple child eating occasions throughout the day; compliance to the EMA protocol outlined in this study was high. We have previously used EMA protocols of similar length in larger samples of more than 600 racially/ethnically and socioeconomically diverse parents in studies using similar EMA protocols [14, 15]. Collectively, these observations provide new insights on real-time use of a wide range of feeding practices and support the feasibility of using the EMA-based Real-Time Parent Feeding Practices survey in observational research with families of young children to improve our understanding of food parenting across time and context.

The parents in the current sample reported the consistent use of a number of structure and autonomy support feeding practices known to be associated with healthful dietary intake outcomes.^1–3,7^ Overall, parents reported the use of one or more structure and one or more autonomy support behaviors at the majority of meals and snacks, respectively. These findings are positive given that the use of structure and autonomy support behaviors have been associated with healthful eating patterns and dietary intake in children in the short and long-term [2, 3, 7]. Parents in this sample reported sitting and eating with their child at more than three fourths of the reported meals and snacks. This finding is notable as research demonstrates that parental presence at meals can benefit children as it provides the opportunity for parents to model mealtime norms and routines as well as the consumption of a wide variety of foods for their child. Understanding the frequency with which parents are present at mealtimes with their preschooler underscores the importance of leveraging parents of young children in interventions that seek to impact the dietary intake or eating patterns of preschoolers. Providing parents with the information they need to best support their child during mealtimes has enormous potential to shape the emerging eating patterns and food preferences of, given the hands-on nature of parenting children at this young age.

The “Division of Responsibility Approach” to feeding, which was first proposed by Ellyn Satter and has since been endorsed by the American Academy of Pediatrics [16] and the Academy for Nutrition and Dietetics, suggests that parents of young children should choose the location of the meal (i.e. where), the timing of meals and snacks (i.e., when) and the specific foods served at the meal (i.e., what) and that children should choose whether they will eat and how much food they will consume at that meal [17]. Examination of the current study findings through the lens of the Division of Responsibility approach helps to highlight ways in which parents align with recommendations and illuminates opportunities for future intervention in the areas where parents are falling short. Specifically, parents reported choosing where their child eats at approximately two-thirds of meals/snacks and choosing what foods are offered at just under a half of meals/snacks. Parents were not asked directly about who chose the timing of the meal/snack. Parents reported having to encourage their child to eat more than they wanted to; having to make sure that their child did not eat too much food; or bribing their child into eating more than they wanted to at 17, 10, and 8% of meals, suggesting that at the majority of, but not all, eating occasions parents allowed their child to take the lead on if they ate or how much food they would eat. Further, parents indicated that they allowed their child to take “seconds” of foods if they asked for them at approximately 65% of meals/snacks, again underscoring that the responsive feeding techniques were used at the bulk of eating occasions. These findings align with, and extend our previous work which sought to understand how parents of preschoolers divide the responsibilities of feeding with their child [18]. In this prior qualitative research, parents gave their child more than the recommended amount of influence over what foods were served and offered children less than the recommended amount of autonomy over the whether and how much or eating. Findings from the present study provide a more nuanced account of the varied approaches parents take to feeding and underscore the importance of continued collaboration by researchers and clinicians to explore alternative frameworks that encourage parents to provide structure and autonomy support, while reducing use of coercive control practices.

In the current study, use of (one or more) indulgent feeding practices was reported by parents at just over one quarter of meals and snacks. The most frequently reported indulgent behavior was parents indicating that they chose to prepare separate food that they knew their child would enjoy eating (reported at 16% of meals/snacks); this finding was notable as this question was developed specifically for the current study based on our prior qualitative work. In this prior research, interviews with parents of preschoolers uncovered a theme of “anticipatory catering”, whereby parents would intentionally purchase and prepare foods that they knew would be well accepted by their child with the goal of avoiding food-related power struggles during meal and snack times. Parents described narrowing their shopping list and meal planning to the foods their child’s favorite foods which they would eagerly consume, thereby avoiding the need to engage in other common food-related parentings practices that would fall under the structure (e.g., rules/routines, limit setting), autonomy support (e.g., negotiation, nutrition education, encouragement), or coercive control (e.g., pressure-to-eat). The current study suggests that this specific type of indulgent feeding practices is used with reasonable frequency among parents of preschool-aged children, highlighting the importance of continuing to explore the extent to which parents of preschool aged children are choosing the foods they serve to their child based solely on their child’s current food preferences. While it makes sense that parents would consider child preferences when shopping for food and planning and preparing meals for their child, the knowledge that preschool aged children are still developing taste preferences and that repeated exposures to new foods is needed to inform taste preferences [4, 19–22], underscore the impact that a parent’s choice to engage in high levels of anticipatory catering might have on the development of their child’s food preferences. Future research is needed to understand the longitudinal impact of anticipatory catering on children’s food preferences and dietary intake.

There are both strengths and limitations to this study. First, this study adds significantly to the emerging literature aimed at broadening our conceptualization of food parenting practices, by being the first, to our knowledge, to measure the broad range of dimensions proposed in the content map developed by Vaughn and other leading experts in the field using EMA. Using novel measures developed for use within an EMA protocol, this study takes a next step towards deepening our understanding of the momentary use of goal-directed food-related parenting practices by enabling researchers to better understand the potential importance of collecting data on these behaviors at the meal-level, rather than relying solely on parents’ one-time survey responses. Future research should extend this work using EMA to examine patterns of food parenting practices, including consistency within meals, variation from meal to meal (or snacks) and across days.

Further, while the overall sample size of this study was small (*n* = 116), the ability to use data from each eating occasion reported via EMA resulted in a total of 3382 data points (i.e., eating occasions) which is a strength of this data collection approach. Data collected using the new EMA-based *Real-Time Parent Feeding Practices* survey is still reliant on parent self-report which may introduce some social desirability bias to responses, however, repetitive, real-time reporting of feeding practices represents a move away from gathering parent report of aspirational perceptions and enables us to capture more variation in behaviors by not asking parents to reduce their actual practices down to a single average response. That said, it is possible that repetitive data collection and reporting on one’s own behavior might act as a mini-intervention, leading parents to change their behavior over the course of the data collection time period.

While the sample was drawn from a large, population-based study and was racially/ethnically and socioeconomically diverse, generalization to other populations should be made cautiously. Further, this sample of parents was highly educated, with over 50% of parents having completed some college or more. Additionally, it is possible that parents who chose to participate in the current study were more interested in their child’s healthful eating as compared to parents who chose not to participate. This information on sample characteristics and potential selection bias is important for readers to consider when interpreting findings. Finally, this manuscript does not provide correlations between this new measurement tool and existing measures, limiting our understanding of how data collected via this instrument compares to similar data collected via previously validated measures; a direct comparison was not made given the hypothesis that this tool measures something different that traditional survey tools. Future research should examine how data reported via the EMA-based *Real-Time Parent Feeding Practices* measurement survey compares to video observation of family meals to understand how parent report of these behaviors correlates with direct observation methods.

## Conclusion

The EMA-based *Real-Time Parent Feeding Practices* survey provides the opportunity to characterize a broad range of food-related parenting practices across time and contexts, in real-time. While supportive feeding practices, including a variety of structure and autonomy support behaviors, were reported most frequently at meals and snacks, findings from the current study suggest that parents engage in a wide range of feeding practices that represent four higher-level domains (e.g., structure, autonomy support, coercive control, indulgence). Importantly, in this study parents reported sitting and eating with their child at the majority of recorded meals, underscoring the potentially important role that parents play in the development of children’s eating habits. Use of EMA to collect data on food-related parenting practices can provide a unique perspective on frequency, variety and patterning of practices used that reaches beyond traditional survey data; the EMA-based *Real-Time Parent Feeding Practices* survey has the potential to inform the development of novel momentary interventions to promote the development of healthful dietary intake and eating patterns in young people. Overall, results from the current study serve to confirm the importance of asking parents about their use of a broad range of food-related parenting practices, rather than inquiring only about practices from one domain (e.g., restriction and pressure-to-eat); for those researchers seeking to engage parents using EMA, the questions used in the current study can serve as a starting point for future inquiry.

## Data Availability

Data and code will be made available upon request and the lead author has full access to the data reported on in the manuscript.
